# Matrix Metalloproteinases in Health and Disease 3.0

**DOI:** 10.3390/biom14091059

**Published:** 2024-08-26

**Authors:** Raffaele Serra

**Affiliations:** 1 Department of Medical and Surgical Sciences, Magna Graecia University of Catanzaro, 88100 Catanzaro, Italy; rserra@unicz.it; 2 Interuniversity Center of Phlebolymphology (CIFL), “Magna Graecia” University, 88100 Catanzaro, Italy

Matrix metalloproteinases (MMPs) are members of an enzyme family that are critical for maintaining tissue allostasis. MMPs can catalyze the normal turnover of the extracellular matrix together with other metalloproteinases, such as the ADAMs (a disintegrin and metalloproteinase) and ADAMTS (a disintegrin and metalloproteinase with thrombospondin motif) families. MMP activity is also regulated by a group of endogenous proteins called tissue inhibitors of metalloproteinases (TIMPs). All these proteins have a pivotal role in normal physiological processes involving extracellular matrix remodeling, such as wound healing, embryogenesis, angiogenesis, bone remodeling, immunity, and the female reproductive cycle. An imbalance in the expression or activity of MMPs can also have important consequences for diseases such as cancer, cardiovascular disease, peripheral vascular disease, chronic leg ulcers, multiple sclerosis, and others [1,2,3,4].

The aim of this Special Issue is to explore the most recent findings in this field that may have an impact on healthcare systems.

In this Special Issue, Zingaropoli et al. [5] showed a positive correlation between TIMP-1 and chest CT scores, thus highlighting its potential use as a marker of fibrotic burden from COVID-19 pneumonia. Plümers et al. [6] showed that MMPs contribute to the calcification phenotype in pseudoxanthoma elasticum, an inherited autosomal recessively genetic defect, dysregulating extracellular matrix remodeling. Skrzypiec-Spring et al. [7] showed that the protective effect of simvastatin on systolic function in the acute ischemia–reperfusion model seems not to be related to reduced MMP-2 activation. Esposito et al. [8] reviewed the role of MMPs in the era of cystic fibrosis transmembrane conductance regulator modulators. Marcianò et al. [9] reviewed all the updated evidence for a possible role of MMPs in pain and the effects of their modulation. Kim et al. [10] reviewed the relationships between MMP and glaucoma. Li et al. [11] reviewed the mechanism and role of the ADAMTS family in osteoarthritis, and they found that ADAMTS may be a potential therapeutic target in osteoarthritis. Bracale et al. [12] conducted a systematic review of the role of matrix metalloproteinases in the pathogenesis of inguinal hernias. Serraino et al. conducted two systematic reviews, one on metalloproteinases and hypertrophic cardiomyopathy [13] and the other on metalloproteinases in cardiac surgery [14], highlighting the importance of the MMP system in cardiovascular disease.

Currently, targeting metalloproteinase regulation and their effects seems to be an evidence-based medicine tool used to counteract multiple pathophysiological mechanisms that underlie a wide range of human diseases [15,16,17,18,19,20,21,22,23].

In conclusion, our efforts should be focused on improving our understanding of the pathophysiological phenomena around metalloproteinases to improve diagnostic and treatment options and to deliver optimal care to patients.
